# Revisiting the Robustness of PET-Based Textural Features in the Context of Multi-Centric Trials

**DOI:** 10.1371/journal.pone.0159984

**Published:** 2016-07-28

**Authors:** Clément Bailly, Caroline Bodet-Milin, Solène Couespel, Hatem Necib, Françoise Kraeber-Bodéré, Catherine Ansquer, Thomas Carlier

**Affiliations:** 1 Nuclear Medicine Department, University Hospital of Nantes, Nantes, France; 2 CRCNA, INSERM, University of Nantes, UMR 892, Nantes, France; 3 Radiology Department, University Hospital of Nantes, Nantes, France; University of Campinas, BRAZIL

## Abstract

**Purpose:**

This study aimed to investigate the variability of textural features (TF) as a function of acquisition and reconstruction parameters within the context of multi-centric trials.

**Methods:**

The robustness of 15 selected TFs were studied as a function of the number of iterations, the post-filtering level, input data noise, the reconstruction algorithm and the matrix size. A combination of several reconstruction and acquisition settings was devised to mimic multi-centric conditions. We retrospectively studied data from 26 patients enrolled in a diagnostic study that aimed to evaluate the performance of PET/CT ^68^Ga-DOTANOC in gastro-entero-pancreatic neuroendocrine tumors. Forty-one tumors were extracted and served as the database. The coefficient of variation (COV) or the absolute deviation (for the noise study) was derived and compared statistically with SUVmax and SUVmean results.

**Results:**

The majority of investigated TFs can be used in a multi-centric context when each parameter is considered individually. The impact of voxel size and noise in the input data were predominant as only 4 TFs presented a high/intermediate robustness against SUV-based metrics (Entropy, Homogeneity, RP and ZP). When combining several reconstruction settings to mimic multi-centric conditions, most of the investigated TFs were robust enough against SUVmax except Correlation, Contrast, LGRE, LGZE and LZLGE.

**Conclusion:**

Considering previously published results on either reproducibility or sensitivity against delineation approach and our findings, it is feasible to consider Homogeneity, Entropy, Dissimilarity, HGRE, HGZE and ZP as relevant for being used in multi-centric trials.

## Introduction

It is well known that tumors often exhibit a broad biological, cellular and tissue heterogeneity [1]. The interactions of cancer cells with their microenvironment are not uniform in the tumor. The conjunctiva-vascular pattern properties that constitute the cancer stroma and the remodelling of the extracellular matrix vary depending on the region of the tumor. Furthermore, local variations in angiogenesis and hypoxia also lead to changes in glucose metabolism [2]. These parameters also determine the aggressiveness of the tumor and its therapeutic resistance. Thus, tumors with a high intrinsic heterogeneity may have a worse prognosis [3].

While 18F-FDG PET images suffer from poor spatial resolution (thus making it difficult to resolve subtle biological process), it is advocated by many that the analysis of tumor heterogeneity by PET may provide useful information for personalized management of disease [4–10]. Based on these assumptions, an increasing number of studies have focused on using PET-based textural features (TFs) as a surrogate biomarker for deriving prognostic and predictive value.

In this context, TFs were first studied in solid cancer, including breast [11], esophageal [12,13], head and neck [14,15], cervical [16] and lung cancers [13,17,18]. The robustness of textural indices was also investigated with respect to their reproducibility [19,20], the choice of discretization value [12,13,21,22], the tumor delineation approach [21,23,24] and the sensitivity to partial volume effect [23]. Many studies have examined the inter-correlation of TFs, and whether they can provide additional information when compared against the standardized uptake value (SUV)-based metrics or volume [13,21,23,24]. The combination of these studies suggests that only a few TFs may be robust enough to be used in a clinical setting. Despite this, it is difficult to derive a set of interesting textural metrics considering the large number and heterogeneity of textural metrics used in each study and the mathematical definition that can be slightly different from one study to another. Additionally, to our knowledge, only two studies reported the robustness of textural indices with respect to acquisition mode and reconstruction parameters [25,26]. Galavis and colleagues considered two iterative reconstruction algorithms with a limited number of lesions, without time-of-flight (TOF) information, without noise consideration and with limited information regarding the discretization value used for computing each textural metric. Yan and colleagues recently published an updated insight based on a current PET system with TOF capability. Indeed, there is a need to re-evaluate the robustness of TFs with respect to reconstruction parameters and to consider the noise level which has not been considered by the aforementioned studies, especially in the context of multi-centric studies which are often retrospective ancillary studies to clinical trials. Under these conditions, PET acquisition and subsequent reconstructions are usually not well controlled although many recommendations have been recently published [27,28]. Additionally, it is well known that a large sample size is required for reducing type-I error and allowing the possibility to separately consider a test dataset for the exploratory analysis and a subsequent validation dataset. This can often only be achieved through a multi-centric study [29].

In this study, our goal was to explore the robustness of few TFs that are clinically investigated by examining their dependence on current reconstruction algorithm, reconstruction parameters (including the number of iterations, the post-filtering properties and the voxel size) and the noise in input data. This study was mainly focused within the framework of multi-centric studies. Hence, different combinations of reconstruction algorithms, related parameters and time per bed position (as a surrogate of noise in input data) were investigated to mimic the conditions encountered in multi-centric studies. Finally, we combined our findings against previously published results sought to study the reproducibility and impact of the delineation approach.

## Materials and Methods

### Population

We retrospectively included a sub-population of 26 patients with proven well differentiated neuroendocrine tumors who were enrolled in a diagnostic multicenter study that aims to evaluate the performance of ^68^Ga-DOTANOC PET/CT in gastro-entero-pancreatic neuroendocrine tumors (https://clinicaltrials.gov/show/NCT01747096). All patients signed a written informed consent form. The median age was 63 years (range, 37–75 y) with 15 men and 11 women. From these 26 patients, 66 tumors confirmed by the gold-standard (all imaging modality and histopathology) were extracted: liver metastases (n = 34), lymph nodes (n = 18), primary lesions (pancreas n = 9; midgut n = 1), bone (n = 1) and carcinomatosis (n = 3).

### PET/CT imaging and reconstruction

The PET/CT scan was performed for all patients using a 4-ring Siemens Biograph mCT system with TOF capability. Patients were injected with 148 ± 16 MBq of ^68^Ga-DOTANOC and scanned for 8 minutes in list mode, 2h after the injection, with one bed position centered on the lesions. The list-mode data from each PET acquisition was truncated to reduce the scan duration to respectively 1 min, 2 min, 3 min and 8 min.

All datasets were reconstructed using 4 different algorithms: 3D attenuation weighted ordered subsets expectation maximization (AW), 3D ordinary Poisson-OSEM in conventional mode (OP), and OP with point-spread function correction (PSF) and TOF mode (PSF-TOF). The default matrix size was 200×200 (voxel size: 4×4×2 mm^3^). Data were also reconstructed using 400×400 (voxel size: 2×2×2 mm^3^) and 256×256 (pixel size: 3.1×3.1×2 mm^3^) depending on the effect studied. Note that reconstructions using a 256×256 matrix were done through an interpolation of the results obtained with the 400×400 matrix. Therefore, reconstructions using a 256×256 matrix were done through an interpolation of the results obtained with the 400×400 matrix.

For each reconstruction algorithm, three different numbers of iterations (2, 4 and 6) combined with three possibilities of full width at half maximum (FWHM) Gaussian post-filtering (all-pass, 2 mm and 5 mm FWHM) were investigated. For sake of clarity, the same number of subsets was used for each algorithm and was set to 24. Hence, for each lesion, the theoretical number of reconstructions was 540, albeit not always used, depending on the impact of acquisition/reconstruction settings on the studied textural features.

### Segmentation and textural features

To minimize the impact of delineation approach on TFs resulting from different reconstruction settings, a unique volume of interest (VOI) for each lesion was delineated on the 200×200 matrix using the OP-OSEM3D+PSF+TOF algorithm based on the 8-min acquisition (default parameters: 2 iterations and 2mm FWHM post-filtering). The VOIs were obtained using an iterative method [30] that involved a calibration specific to the system used. When required, these initial VOIs were interpolated (bi-cubic) to larger matrix size (256×256 or 400×400) with the constraint that the interpolated volume must be within 1% of the initial volume. Finally, all lesions larger than 2 cm^3^ (64 voxels) were included in the subsequent analysis [16,21]. This narrowed the number of lesions studied to 41, with a volume of 17.4 ±34.1 cm^3^ (range: 2.2–179.7 cm^3^).

The TFs we chose to study are among the most widely used in recent publications. We mainly focused on metrics where a test-retest reproducibility study had already been conducted [19,20]. As such, 6 TFs were extracted from the grey level co-occurence matrix (GLCM), 3 TFs from the grey level run length matrix (GLRLM) and 6 from the grey level size zone matrix (GLSZM). The GLCM and GLRLM were calculated from 13 directions with one-voxel displacement. The final TF was computed by averaging TFs over the 13 directions. A list of all the TFs studied along with the mathematical definition is provided in S1 Table. The SUV values within each VOI were resampled using 64 discrete values [13]. Finally, two first-order parameters were also derived from the VOI to be compared with TFs: SUVmax and SUVmean.

### Study design

Each reconstruction setting was studied by making all other parameters constant in order to correctly individualize the impact of each investigated parameter. The influence of matrix size was studied for only one algorithm (PSF-TOF) using the default reconstruction parameters (2 iteration, post-filtering 2 mm FWHM). However, in order to fully decorrelate the impact of matrix size from the noise in input data, the time for the largest matrix size (400×400) was adapted to match noise properties found for the original matrix size (200×200). In this situation, a cylinder of ^68^Ge was acquired during 120 s and reconstructed using the default reconstruction settings. The signal-to-noise ratio (SNR), defined as the standard deviation over the mean measured in a uniform region was then derived. A second acquisition of the same phantom was then acquired in list-mode and reconstructed with the same reconstruction settings except the matrix size (400×400). The acquisition time that led to an identical SNR from the one obtained from the 200×200 (120 s used) was 188 s. This duration was also set for reconstructing data using a 256×256 matrix size as outlined above. Table 1 lists the different reconstruction configurations used for studying the relative dependence of reconstruction parameters on TFs.

**Table 1:** List of the different reconstruction parameters used as a function of the parameters studied.

Parameters studied	Range	Constant parameters
**Number of iterations**	2, 4 and 6	Post-filtering (0 mm FWHM) and time (180 s)
**Level of post-filtering (mm FWHM)**	0, 2 and 5	Number of iterations (2) and time (180 s)
**Noise (acquisition time in s)**	60, 120 and 180	Number of iterations (2) and post-filtering (2 mm FWHM)
**Reconstruction algorithm**	AW, OP, PSF and PSF-TOF	Number of iterations (2), post-filtering (0 mm FWHM) and time (180 s)
**Matrix size**	200×200, 256×256 and 400×400	PSF-TOF (2 iterations and 2 mm FWHM post-filtering) using 120 s (200×200) or 188 s (256×256 and 400×400)

**Fig 1:**
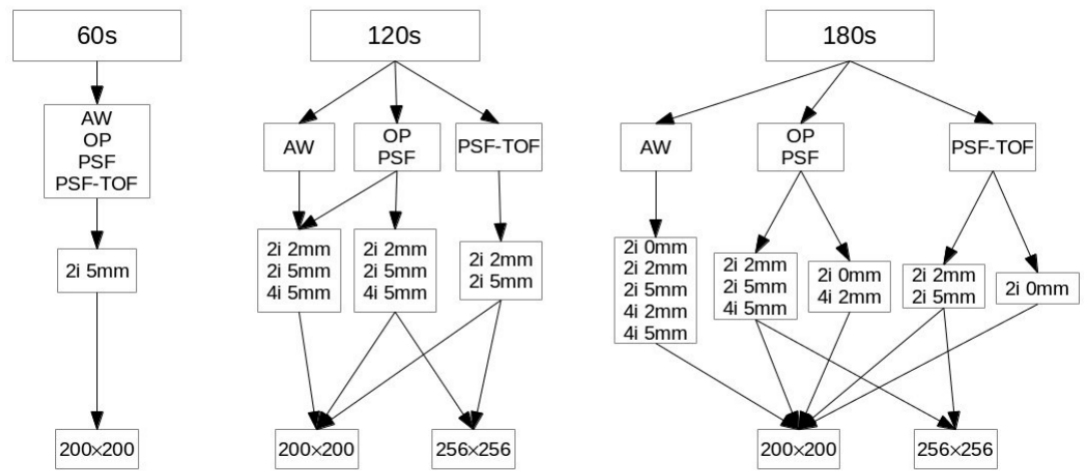
Acquisition and reconstruction settings. List of each acquisition setting (defined by the time considered) with the reconstruction algorithm and attached parameters for mimicking conditions encountered in multi-centric trials. “i” represents the number of iterations, “mm” the FWHM Gaussian post-filtering and 200×200 or 256×256 the matrix size used.

Finally, a subset of reconstruction parameters that are very similar in terms of acquisition time, number of iterations, post-filtering level and matrix size were examined, as they mimic conditions found in an on-going multi-centric trial [24], and are applicable to multi-centric trials. Obviously, it was not possible to use exactly the same algorithms as found in the multi-centric trial because different PET systems and attached reconstruction algorithms were used. Hence, we tried to cover most of the parameters used in the multi-centric trial under the assumption that the difference that can be found in the original data extracted from the multi-centric trial can be “simulated” by selecting a subset of different parameters chosen among our algorithms and attached parameters. This led for each lesion, to a total of 49 different reconstructions whose configurations are listed in Fig 1.

### Metrics

The coefficient of variation (*COV*^*L*^) was the metric used to analyze the dependence of TFs for all investigated parameters except noise. *COV*^*L*^ was also used when considering a combination of different reconstruction settings (detailed hereafter) to mimic multi-centric conditions. The *COV*^*L*^ calculated for each lesion *L* was defined by:

$$COV^{L}=\text{100}\times \frac{\sqrt{\frac{\text{1}}{N-\text{1}}\overset{N}{\underset{k=\text{1}}{\sum }}{\left(m_{k}^{L}-{\bar{m}}^{L}\right)}^{\text{2}}}}{{\bar{m}}^{L}}$$ (1)

where $m_{k}^{L}$ is the measurement of TFs (including SUVmax and SUVmean) for lesion *L* related to the metrics analyzed and ${\bar{m}}^{L}$ is the mean value of lesion *L* over the *N* measurement. By definition, *N* = 3 for the study related to the impact of the number of iterations, post-filtering level or matrix size, *N* = 4 for the study related to the impact of reconstruction algorithm and *N* = 49 when combining a different set of reconstruction parameters for the multi-centric-like study.

The impact of noise in the input data was investigated by computing the percentage deviation *D*^*L*^ of the TF for each lesion *L* related with the 8-min acquisition defined as the gold standard, using:

$$D^{L}=\text{100}\times \frac{\sqrt{\frac{\text{1}}{N-\text{1}}\overset{N}{\underset{k=\text{1}}{\sum }}{\left(t_{k}^{L}-{\bar{t}}^{L}\right)}^{\text{2}}}}{{\bar{t}}^{L}}$$ (2)

where

$$t_{k}^{L}=\text{100}\times \frac{m_{k}^{L}-m_{\text{480}}^{L}}{m_{\text{480}}^{L}}$$ (3)

$m_{k}^{L}$ is the measurement of TFs for lesion *L* and for a time *k* expressed in seconds (*k*ϵ[60;120;180]), $m_{\text{480}}^{L}$ is the TF value for the acquisition time of 480 s and ${\bar{t}}^{L}$ is the mean value of lesion *L* over the *N* measurement.

Finally, as SUVmax and, to a lesser extent, SUVmean are the most used quantitative parameters in multi-centric trials, the robustness of each TF was ranked against them relatively (Table 2) as also suggested by Buvat and colleagues [31]. For this purpose, each TF was compared to both SUVmax and SUVmean using a one-way ANOVA for repeated measures with the Tukey HSD test. A Bonferroni correction was applied for multiple comparison testing.

**Table 2:** Classification of the TF robustness with respect to SUV-based robustness.

Rule	Robustness
*M*_*TF*_ not statistically different from *M*_*SUVmean*_ *M*_*TF*_ statistically different from *M*_*SUVmax*_ with *M*_*TF*_ < *M*_*SUVmax*_	High
*M*_*TF*_ not statistically different from *M*_*SUVmax*_	Intermediate
*M*_*TF*_ statistically different from *M*_*SUVmax*_ with *M*_*TF*_ > *M*_*SUVmax*_	Low

*M* stands for the metrics used (COV or D).

## Results

### Impact of number of iterations, level of post-filtering, reconstruction algorithm and noise in input data

**Fig 2:**
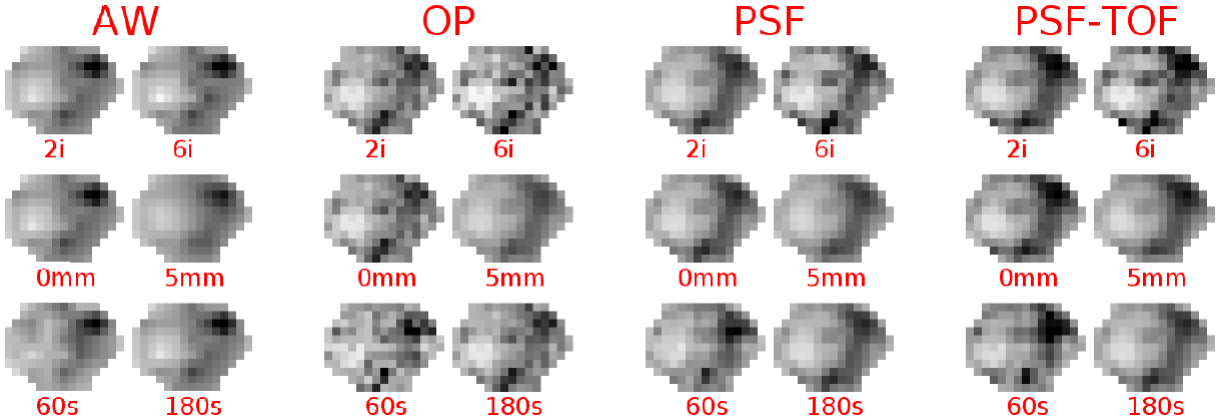
Tumor illustration. Illustration of a tumor (axial slice) reconstructed using different reconstruction settings. Two different images are presented for each reconstruction algorithm studied (AW, OP, PSF and PSF-TOF) corresponding to the minimum and maximum value of the parameters investigated (number of iterations, level of post-filtering and acquisition time). Upper row: variation of the number of iterations (2 and 6 iterations). Middle row: variation of the post-filtering level (0 mm or 5 mm FWHM). Bottom row: variation of the acquisition time for a surrogate of noise in the input data (60 s or 180 s). The grey scale level is identical for each image.

Fig 2 gives an example of the impact of several acquisition/reconstruction settings on the final image for a heterogeneous lesion (volume: 124.6 cm^3^). This example highlights the difference in the heterogeneity pattern that can be met when considering several sets of reconstruction parameters.

**Fig 3:**
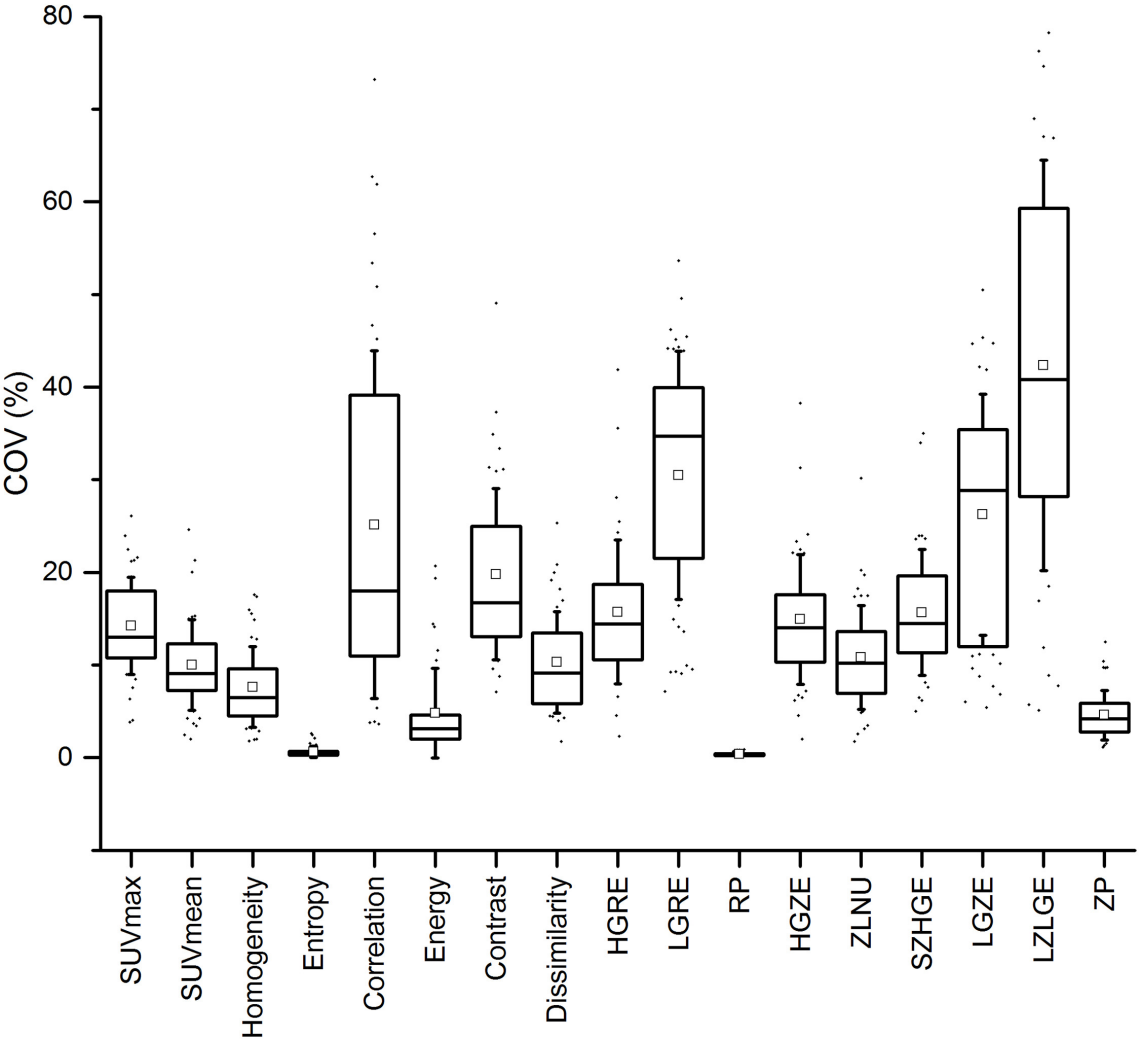
Impact of the reconstruction algorithm. Impact of the reconstruction algorithm on each TF using the default settings outlined in Table 1.

The complete results describing the impact of the number of iterations, the level of post-filtering and the noise in the input data are presented in S1–S3 Figs. The impact of the reconstruction algorithm is shown in Fig 3, while a summary of these results is presented in Table 3 relative to the results of SUV-based metrics as explained in Table 2. To clarify, Table 3 shows only the results for the PSF-TOF algorithm (except when considering the impact of reconstruction algorithm wherein the four algorithms were used) given that results were generally found to be similar for the 3 other algorithms.

**Table 3:** Robustness of each TF with respect to the robustness of SUV-based metrics.

Robustness	High	Intermediate	Low
**Number of iterations**	Homogeneity, Entropy, Energy, Dissimilarity, RP, ZP	Contrast, HGRE, HGZE, ZLNU, SZHGE	Correlation, LGRE, LGZE, LZLGE
**Post-filtering level**	Homogeneity, Entropy, Energy, Contrast, Dissimilarity, RP, ZP	Correlation, HGRE, LGRE, HGZE, ZLNU, SZHGE, LGZE	LZLGE
**Noise**	Entropy, Energy, RP, ZP	Homogeneity, Dissimilarity, ZLNU	Correlation, Contrast, HGRE, LGRE, HGZE, SZHGE, LGZE, LZLGE
**Reconstruction algorithm**	Homogeneity, Entropy, Energy, Dissimilarity, RP, ZP	HGRE, HGZE, ZLNU, SZHGE	Correlation, Contrast, LGRE, LGZE, LZLGE

The impact of the number of iterations, the post-filtering level and the noise in input data were for the PSF-TOF algorithm. The impact of the reconstruction algorithm was derived using the 4 algorithms available (AW, OP, PSF and PSF-TOF).

Among the TF studied in this work, 4 (Entropy, Energy, RP and ZP) were found to be robust enough against the number of iterations, the post-filtering level, the noise in input data and the reconstruction algorithm with respect to results related to SUVmax and SUVmean. Homogeneity and Dissimilarity presented very similar properties apart from their robustness against noise which was found to be intermediate. In contrast, 3 TFs displayed the poorest performance (Correlation, LGZE and LZLGE) among all investigated parameters (except the post-filtering level for Correlation). HGRE, ZLNU, HGZE and SZHGE yielded intermediate robustness except for the noise in input data where HGRE, HGZE and SZHGE were found to be more sensitive to noise than SUVmax. Finally, Contrast and LGRE performed equally with a low robustness with respect to noise and reconstruction algorithm and an intermediate robustness when considering respectively the number of iterations and the post-filtering level.

The results derived from the PSF-TOF algorithm (except when considering the impact of reconstruction algorithm) remained valid for the three other algorithms except for the impact of the number of iterations with the AW algorithm. In this particular case, most of the TFs, that exhibited high robustness using either OP, PSF of PSF-TOF, showed an intermediate robustness (except Entropy and RP which performed equally). Similarly, a low robustness was found for those that previously reached an intermediate robustness.

### Impact of matrix size

**Fig 4:**
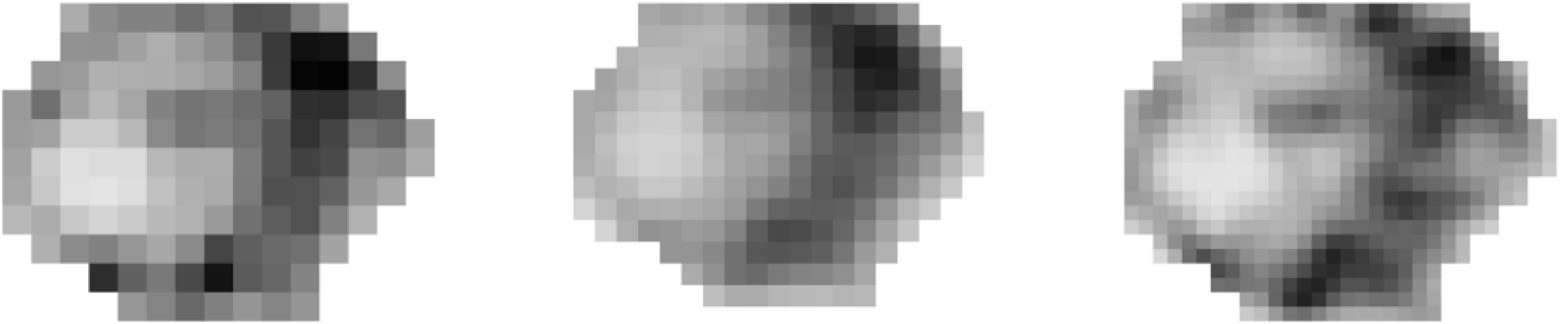
Impact of the matrix size. Impact of the matrix size used for reconstruction (PSF-TOF with 2 iterations and 2 mm FWHM Gaussian post-filtering). Left: 200×200 (voxel size: 4×4×2 mm^3^), middle: 256×256 (voxel size: 3.1×3.1×2 mm^3^), right: 400×400 (voxel size: 2×2×2 mm^3^). The grey scale level is identical for each image.

Fig 4 illustrates the differences observed when reconstructing with a voxel size of 4×4×2 mm^3^ (matrix size: 200×200) or 2×2×2 mm^3^ (matrix size: 400×400).

**Fig 5:**
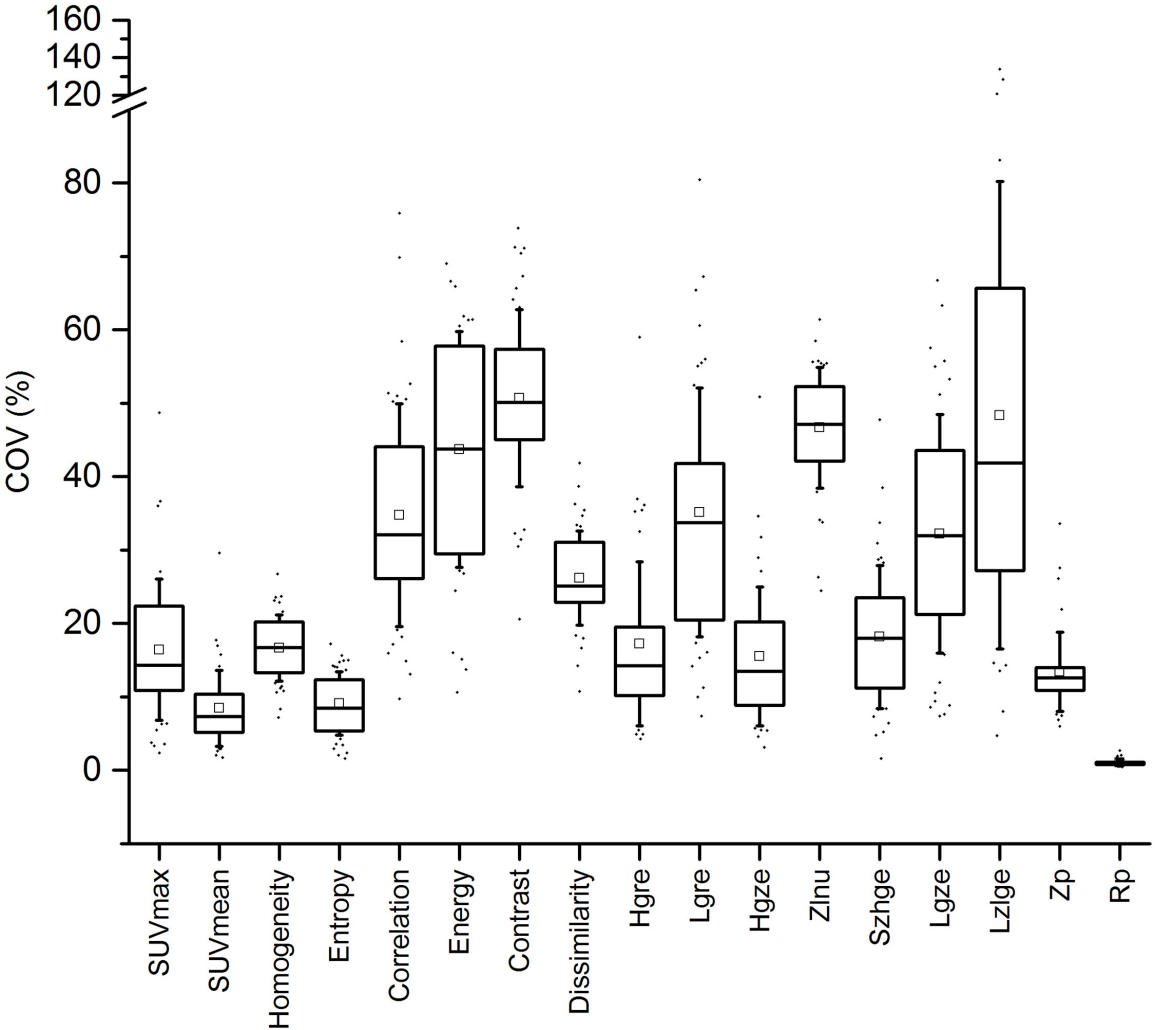
Impact of the matrix size using PSF-TOF.

Fig 5 shows the variation of the COV for each TF and SUV-based metrics while Table 4 summarizes the TF robustness with respect to SUV-based results. The voxel size has a strong impact on the robustness of TFs as only 4 of them exhibited a small variability (equivalent or less than SUVmean). All other studied metrics showed an intermediate (Homogeneity, HGRE, HGZE, SZHGE, ZP) or large variation (Correlation, Energy, Contrast, Dissimilarity, LGRE, ZLNU, LGZE, LZLGE) with respect to SUV.

**Table 4:** Robustness of the matrix size. Robustness of each TF with respect to the robustness of SUV-based metrics as a function of the matrix size for the PSF-TOF algorithm.

Robustness	High	Intermediate	Low
**Matrix size**	Entropy, RP	Homogeneity, HGRE, HGZE, SZHGE, ZP	Correlation, Energy, Contrast, Dissimilarity, LGRE, ZLNU, LGZE, LZLGE

### Combination of multiple reconstruction algorithms

**Fig 6:**
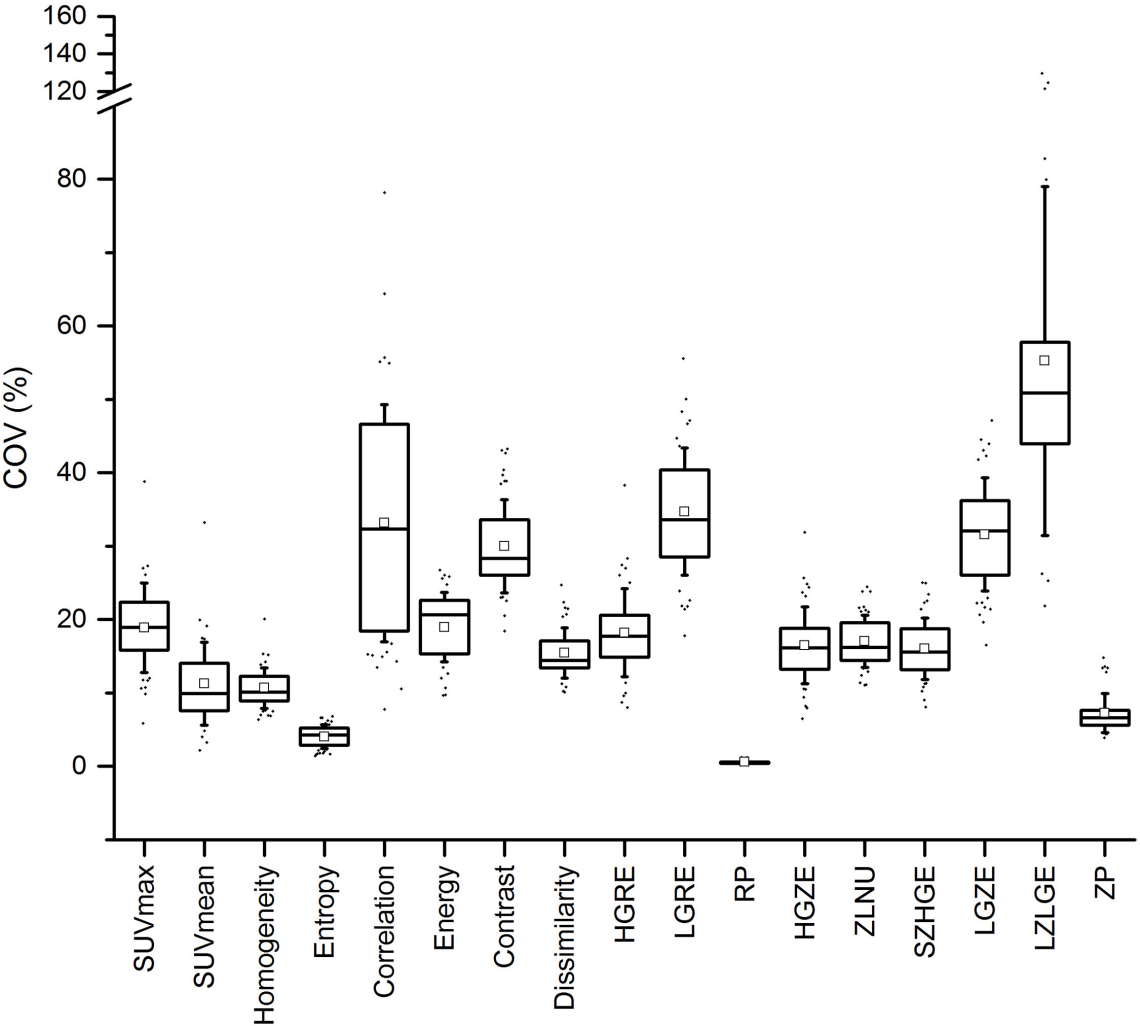
Robustness for multi-centric conditions. Variation of the COV for each TF when pooling different reconstructions detailed in Table 1 in order to mimic multi-centric conditions.

Fig 6 illustrates the COV variability of each metric while Table 5 summarizes the final robustness with respect to SUV-based metrics.

**Table 5:** Robustness with combination of multiple parameters. Robustness of each TF with respect to the robustness of SUV-based metrics when combining multiple parameters (see details in Fig 1)

Robustness	High	Intermediate	Low
**Combination of multiple parameters**	Homogeneity, Entropy, RP, ZP	Energy, Dissimilarity, HGRE, HGZE, ZLNU, SZHGE	Correlation, Contrast, LGRE, LGZE, LZLGE

Seven TFs appeared to be robust enough in this context (Homogeneity, Entropy, RP and ZP) while 5 others are not advisable for use within multi-centric trials. Energy, Dissimilarity, HGRE, HGZE, ZLNU and SZHGE presented intermediate results.

## Discussion

Since the first application of heterogeneity analysis derived from PET images by El Naqa [32], the assessment of TF as a prognostic bio-marker has gained increasing interest mainly in the context of solid tumors [11–16,18,23] and marginally for haemopathies [33,34]. However, there is still a need for validating the potential interest of TF with large prospective cohorts in order to minimize type-I error using a validation dataset [29]. This requirement can be adequately fulfilled within the framework of multi-centric studies. In this situation, it is well known that different PET systems and associated reconstruction settings may lead to different textured noise, contrast and resolution [28,35] which may impair in turn the robustness of TF analysis. The variability of several TF as a function of reconstruction algorithm (iterative algorithm without PSF correction nor TOF information) and acquisition mode (2D or 3D) was devised [25] and reported interesting results which are still used in several studies to select the best TF metrics. However, we did not attempt to compare our results with those of Galavis and colleagues [25] for several reasons. Briefly, no information could be found about the resampling strategy that is known to impact the final results [21,22,26]. Also, the definition of each TF metric was not reported although this is now established that a same name is not synonym of an identical mathematical definition and hence could led very different results [31]. Finally, there were no details about the lesion volumes (and mostly the number of voxels included) which makes difficult a robust comparison with our results. These initial results were recently updated with the use of reconstruction algorithms that take advantage of PSF corrections combined (or not) with TOF information [26].

In this study, we focused on the variability of TF using different settings of current reconstruction algorithms within the framework of multi-centric trials. In this respect, our study differed from the work of Yan & colleagues for several reasons. The impact of noise in input data was carefully investigated. This may be particularly interesting given that the sensitivity of different PET systems may differ and obviously the acquisition time per bed position is rarely the same between centers. The variability of each TF was also investigated against the reconstruction algorithm used. For this purpose, we considered an algorithm (AW) that yielded a textural pattern very different from the ordinary Poisson based algorithm (see Fig 2) which could account for the use of older PET systems and associated reconstruction algorithms. The robustness of each TF was also assessed against SUV-based metrics with a combination of multiple reconstruction settings (Fig 1) that may represent the variability met when several centers are part of a large clinical study. The choice of the different reconstruction settings was inspired by the conditions found in an on-going multi-centric trial on mantle cell lymphoma [24,32]. Additionally, we included two times more lesions than the two other previously published studies on this topic [25,26]. The number of TF considered in this current study was limited to those that were previously studied against reproducibility [19,20] as this property is essential when assessing new quantitative metrics. We also investigated the impact of matrix size through the use of three different voxel sizes with the aim of de-correlating the impact of noise from the impact of voxel size. These two correlated effects (matrix size and noise) were not previously accounted for in an independent manner. Finally, each TF was ranked against SUVmax and SUVmean using the whole variability of the COV and not only the mean or the minimum and maximum values.

In this study, we showed that among the investigated TF only a few of them appeared robust enough with respect to the number of iterations, the post-filtering level, the noise in input data and the reconstruction algorithm used: Entropy, Energy, RP and ZP. In contrast, Correlation and LZLGE were found to be very sensitive to the aforementioned parameters and should be discarded when considering their use in a multi-centric context. The remaining TFs investigated were divided between those with a high/intermediate robustness (Homogeneity, Dissimilarity and ZLNU) and an intermediate/low robustness (HGRE, LGRE, HGZE, LGZE and SZHGE). Among this last category, HGRE, HGZE and SZHGE presented an intermediate variability (equivalent to SUVmax) for the number of iterations, the post-filtering level and the reconstruction algorithm. Thus, based solely on those individual results, in the sense of not being combined, most of the investigated TFs can be used in a multi-centric context except Correlation, Contrast, LGRE, LGZE and LZLGE. These results were approximately in line with those found by Yan & colleagues with, however, noticeable differences for LGRE and LGZE (high vs low COV with respect to the number of iterations for respectively, our study and their results) and ZP (low vs high COV with respect to the number of iterations and the post-filtering level for respectively, our study and their results). Whilst no mathematical definitions were provided in the work of Yan and colleagues, we first hypothesized that these discrepancies were likely due to a difference of number of voxels taken into account in the computation. For this purpose, we attempted to select tumors with a number of voxels similar with values reported in the work of Yan & colleagues. We ended up with 15 tumors (781 ± 809 voxels; range: 271–3289) that can be seen as roughly identical to the number of voxels used by Yan et al (737 ± 860 voxels; range: 102–3133). The impact of the number of iterations was re-assessed, but our results did not change for LGRE, LGZE and ZP although we hypothesize that the same reconstruction parameters were used (no details provided in the work of Yan et al when reporting individual results related to each reconstruction parameters). It is thus very difficult to derive a plausible explanation without making assumptions that mathematical definitions and implementations were different.

The voxel size used for reconstructing PET images had a large detrimental impact for Correlation, Energy, Contrast, Dissimilarity, LGRE, ZLNU, LGZE and LZLGE. Only, Entropy and RP presented a variability equal to or less than that of SUVmean. The remaining TFs displayed an intermediate robustness. Our results were very different from those found by Yan & colleagues for all TF except Entropy. For example, they found LGRE, HGRE and LGZE very robust (COV<5%) whilst the robustness of these parameters was low in our study (COV larger than the COV of SUVmax and COV>17%). In contrast, the robustness of RP was low in their study and high in ours. The mean COV of LZLGE was between 10% and 20% for Yan & colleagues and more than 49% in our work. These marked differences may be partly explained by the fact that we considered two times more lesions, three voxel sizes rather than two (from 8 mm^3^ to 32 mm^3^ for our present study vs 48 mm^3^ and 192 mm^3^) and we de-correlated the impact of noise by adapting the statistical property of the largest matrix size to the smallest one. Indeed, the impact of noise in the input data for LGRE, HGRE and LGZE was found to be significant in our study (S3 Fig) and can also partly explain the difference with previously published results if noise was not taken into account when deriving the impact of matrix size.

Finally, we combined multiple reconstruction and acquisition settings so that conditions met in multi-centric trial may be simulated. The conclusions drawn when considering each parameter individually were not changed for the majority of the TF studied. In this respect, Homogeneity, Entropy, RP and ZP presented a variability equivalent to or lower than that of SUVmean. Hence, these metrics seem to be suitable for use in a multi-centric context. Dissimilarity and Energy were very sensitive to the matrix size and were subsequently ranked as intermediate whereas they were found to be robust when considering the other parameters (except noise for Dissimilarity). In the same category (intermediate), HGRE, HGZE, ZLNU and SZHGE showed variability similar to SUVmax. These metrics can also be good candidates within a multi-centric context given the same variability of the most used quantitative metrics (SUVmax). In contrast, Correlation, Contrast, LGRE, LGZE and LZLGE should be avoided for their high variability with respect to SUVmax. This last conclusion contradicts the findings of Yan & colleagues for at least LGRE and LGZE. The same holds true for ZP which was previously found to be less robust than SUVmax whilst this metric presented a high robustness given our findings. As stated earlier, this discrepancy cannot be easily explained for LGRE, LGZE and ZP. However, an additional analysis was also conducted to address the issue of dependence of TF with respect to volume. Two sub-populations were chosen (<10 cm^3^ and >10 cm^3^) [13] keeping only data reconstructed with the 200×200 matrix size. No significant differences were found (data not shown) between the two sub-populations (except for ZLNU and Correlation) suggesting that the conclusions remain valid regardless of tumor volume for a same voxel size.

This work has several limitations. We evaluated the robustness of each TF using reconstruction algorithms developed by only one manufacturer. However, we believe that the algorithms investigated in this study presented enough difference to be considered as a valid alternative to assess the TFs variability with different implementation of reconstruction algorithms. We also used data obtained from patients enrolled in a clinical trial that aimed to assess the potential of PET/CT ^68^Ga-DOTANOC in the exploration of well-differentiated gastro-enteropancreatic neuroendocrine tumors. The positron range of ^68^Ga is larger than ^18^F which may potentially impair the translation of those conclusions to ^18^F-FDG. However, given the voxel size currently used in clinical conditions, we assumed that this effect had a limited effect on textured pattern.

Finally, it is possible to link the conclusions reported in this study with those drawn by others that were focused on reproducibility [19,20] and sensitivity to the segmentation approaches [21,23,24]. Combining these different results lead to 6 potentially interesting TFs: Homogeneity, Entropy, Dissimilarity, HGRE, HGZE and ZP. These identified metrics will be assessed prospectively in an on-going multi-centric trial on mantle cell lymphoma [24,33]. It is worth noting that the correlation between these TFs must be considered as many of them can provide the same information [13,24].

## Conclusions

In this study, we estimated the robustness of textural features within the framework of multi-centric trials. We analyzed the dependence using various reconstruction settings and by combining several of them. We showed that only a few of them, including Homogeneity, Entropy, Dissimilarity, HGRE, HGZE and ZP, presented a variability similar to or less than SUVmax.

## Supporting Information

S1 Fig Impact of the number of iterations. Impact of the number of iterations on TF for the 4 reconstruction algorithms considered (AW, OP, PSF and PSF-TOF).
(JPG)

S2 Fig Impact of the post-filtering level. Impact of the post-filtering level on TF for the 4 reconstruction algorithms considered (AW, OP, PSF and PSF-TOF).
(JPG)

S3 Fig Impact of noise. Impact of noise in input data on TF for the 4 reconstruction algorithms considered (AW, OP, PSF and PSF-TOF).
(JPG)

S1 Table Mathematical definitions of each textural feature. (DOC)
