# Supplementary figures and images for: Revisiting the Robustness of PET-Based Textural Features in the Context of Multi-Centric Trials
Source: PLoS One. 2016 Jul 28;11(7):e0159984. doi: 10.1371/journal.pone.0159984 (PMC4965162; doi:10.1371/journal.pone.0159984)

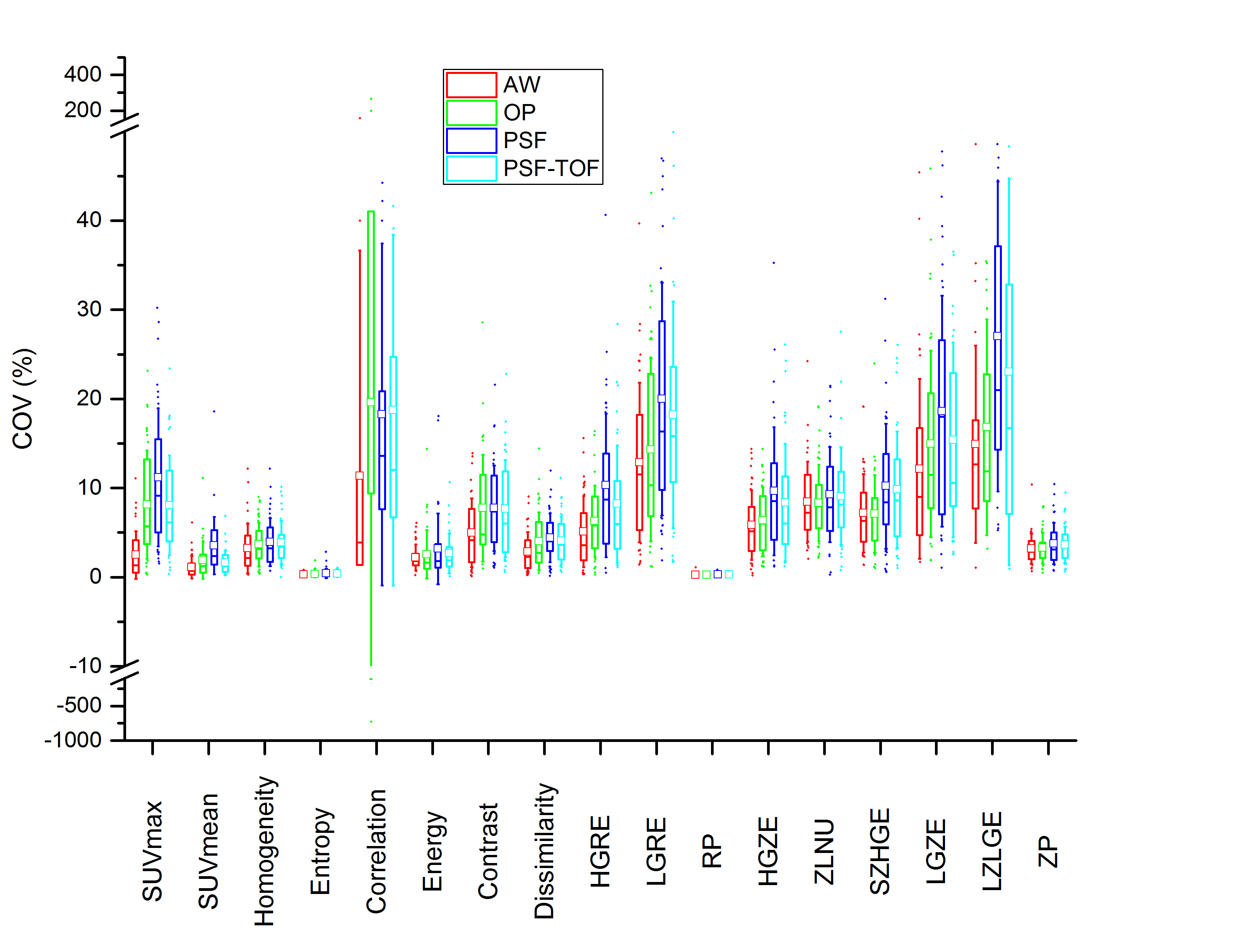

Supplement: S1 Fig — Impact of the number of iterations on TF for the 4 reconstruction algorithms considered (AW, OP, PSF and PSF-TOF). (JPG) [file pone.0159984.s001.jpg]

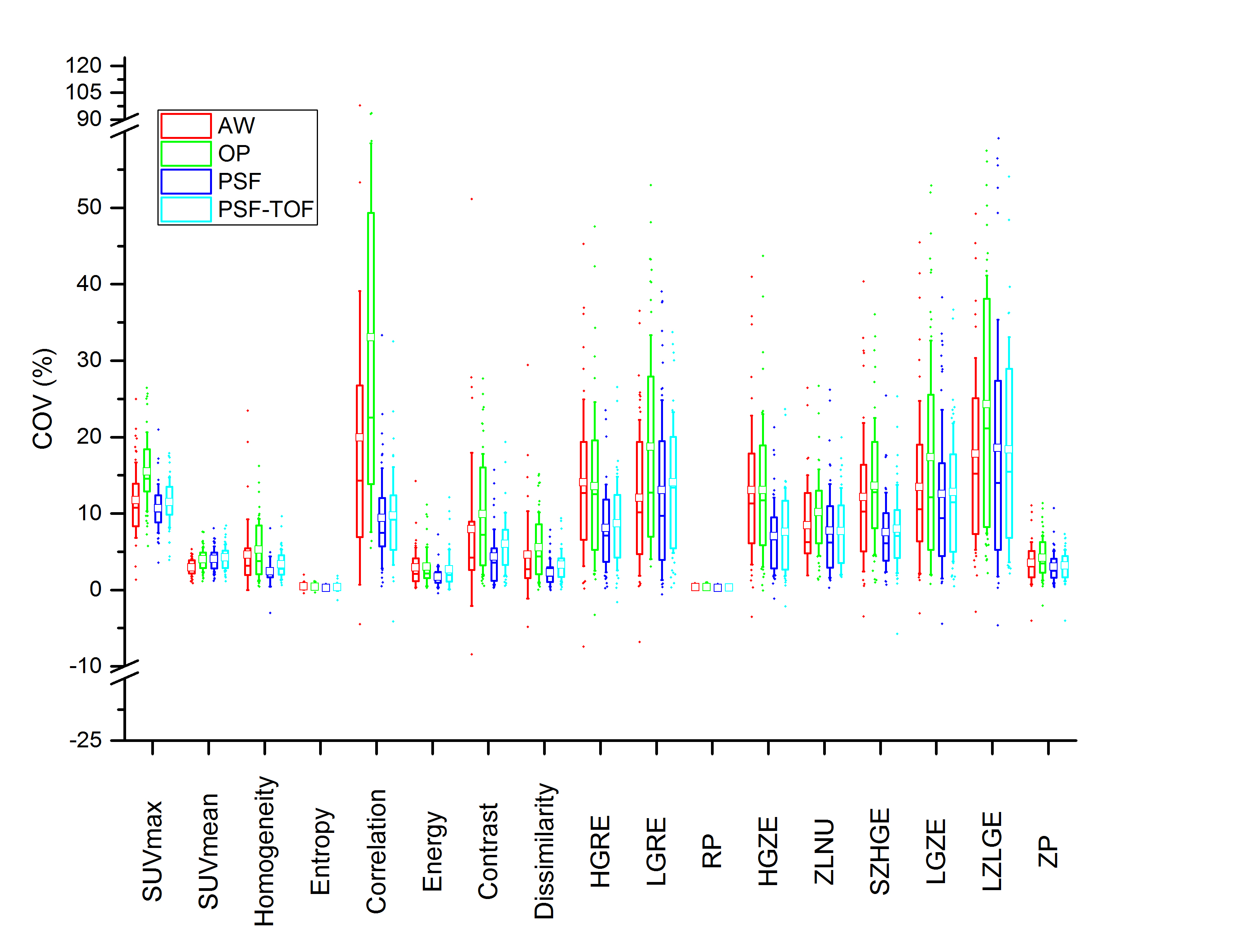

Supplement: S2 Fig — Impact of the post-filtering level on TF for the 4 reconstruction algorithms considered (AW, OP, PSF and PSF-TOF). (JPG) [file pone.0159984.s002.jpg]

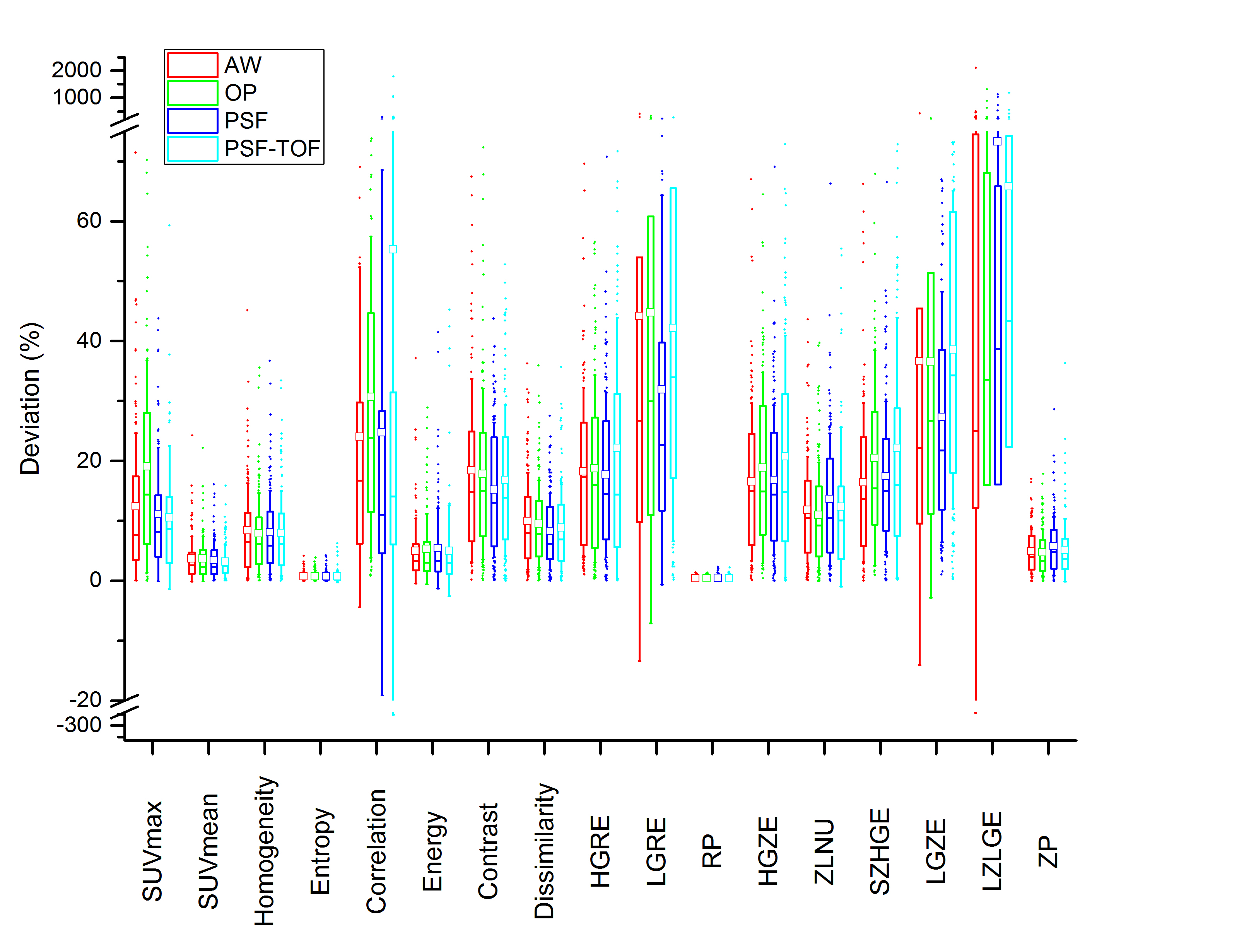

Supplement: S3 Fig — Impact of noise in input data on TF for the 4 reconstruction algorithms considered (AW, OP, PSF and PSF-TOF). (JPG) [file pone.0159984.s003.jpg]
