# Supplementary material for: Revisiting the Robustness of PET-Based Textural Features in the Context of Multi-Centric Trials
Source: PLoS One. 2016 Jul 28;11(7):e0159984. doi: 10.1371/journal.pone.0159984 (PMC4965162; doi:10.1371/journal.pone.0159984)
Supplement: S1 Table — (DOC) [file pone.0159984.s004.doc]

| **Matrix name** | **Textural feature name** | **Equation** | **Description** |
| --- | --- | --- | --- |
| **Grey level co-occurence matrix (GLCM)** | Homogeneity | $\sum_{i} \sum_{j} \frac{C_{ij}}{1+\left\vert i-j\right\vert}$ | Local homogeneity of paired voxels |
|  | Entropy | $-\sum_{i} \sum_{j} C_{ij}\times\text{log}C_{ij}$ | Local measure of information content |
|  | Correlation | $\sum_{i} \sum_{j} \left(i-\mu_{i}\right)\left(j-\mu_{j}\right)\frac{C_{ij}}{\sigma_{i}\sigma_{j}}$ | Measure of linear dependency between intensities  of paired voxels |
|  | Energy | $\sum_{i} \sum_{j} C_{ij}^{2}$ | Uniformity of intensity |
|  | Contrast | $\sum_{i} \sum_{j} (i-j)^{2}C_{ij}$ | Local intensity variations |
|  | Dissimilarity | $\sum_{i} \sum_{j} \vert i-j\vert\times C_{ij}$ | Linear weighted contrast |
| **Grey level run length matrix (GLRLM)** | High Grey Level Run Emphasis (HGRE) | $\frac{1}{\Omega}\sum_{i} \sum_{j} R_{ij}\times i^{2}$ | Distribution of high grey-level run |
|  | Low Grey Level Run Emphasis (LGRE) | $\frac{1}{\Omega}\sum_{i} \sum_{j} \frac{R_{ij}}{i^{2}}$ | Distribution of low grey-level run |
|  | Run Percentage (RP) | $\frac{\Omega}{\sum_{i} \sum_{j} j\times R_{ij}}$ | Percentage of run |
| **Grey level size zone matrix (GLSZM)** | High Grey-level Zone Emphasis (HGZE) | $\frac{1}{\Omega}\sum_{i} \sum_{j} Z_{ij}\times i^{2}$ | Distribution of high grey-level zone |
|  | Zone Length Non Uniformity (ZLNU) | $\frac{1}{\Omega}\sum_{j} \left[\sum_{i} Z_{ij}\right]^{2}$ | Measure of size zone variability |
|  | Short-Zone High Gray-level Emphasis (SZHGE) | $\frac{1}{\Omega}\sum_{i} \sum_{j} \frac{Z_{ij}\times i^{2}}{j^{2}}$ | Distribution of small zone of high grey-level |
|  | Low Gray-level Zone Emphasis (LGZE) | $\frac{1}{\Omega}\sum_{i} \sum_{j} \frac{Z_{ij}}{i^{2}}$ | Distribution of low grey-level zone |
|  | Long-Zone Low Gray-level Emphasis (LZLGE) | $\frac{1}{\Omega}\sum_{i} \sum_{j} \frac{Z_{ij}\times j^{2}}{i^{2}}$ | Distribution of large zone of low grey-level |
|  | Zone Percentage (ZP) | $\frac{\Omega}{\sum_{i} \sum_{j} j\times Z_{ij}}$ | Percentage of zone |

Where:

- *C_ij_* is the co-occurence matrix
- $\mu_{i}$, $\mu_{j}$, $\sigma_{i}$and $\sigma_{j}$are respectively the means of row *i* and column *j* and the standard deviations of row *i* and column *j*
- *R_ij_* is the GLRL matrix
- *Z_ij_* is the GLSZ matrix
- $\Omega$is the number of runs or zones
